# Play Elements as Mechanisms in Intergenerational Arts Activities to Support Community Engagement with End-of-Life Issues

**DOI:** 10.3390/healthcare9060764

**Published:** 2021-06-19

**Authors:** Max Kleijberg, Rebecca Hilton, Beth Maina Ahlberg, Carol Tishelman

**Affiliations:** 1 Department of Learning, Informatics, Management and Ethics, Karolinska Institute, 117 11 Stockholm, Sweden; carol.tishelman@ki.se; 2 Research Centre, Stockholm University of the Arts, 104 50 Stockholm, Sweden; rebecca.hilton@uniarts.se; 3 Skaraborg Institute for Research and Development, 541 30 Skövde, Sweden; beth.maina.ahlberg@kbh.uu.se; 4 Department of Sociology, Uppsala University, 751 26 Uppsala, Sweden; 5 Centre for Health Economics, Informatics and Health Care Research, Stockholm Health Care Services (SLSO), Region Stockholm, 171 11 Stockholm, Sweden

**Keywords:** end-of-life, health promotion, arts activities, community-based programs, children, older people, intergenerational, play

## Abstract

Talking about dying, death, and loss may be difficult. Arts offer alternative ways of engaging with end-of-life (EoL) issues, but little is known about the means through which this occurs. In this article, we aim to explore mechanisms in arts activities that support community engagement with EoL issues, based on the community-based participatory action research project Studio DöBra. Studio DöBra was developed to support community engagement with EoL issues through intergenerational arts workshops involving community partners, children, and older adults. Initial analysis with community partners indicated the importance of play elements in arts activities. Continued analysis was therefore abductive, using play theory and qualitative data from Studio DöBra arts activities. Through iterative examination of theory and data, we modified play theory as we identified mechanisms supporting community engagement with EoL issues in arts activities. Findings can contribute to theory-building that can inform arts activities supporting community engagement with EoL issues.

## 1. Introduction and Aim

Although death and loss are all around us—in our private lives, in the news, and other media—talking about these issues may be difficult as they are often considered sensitive and involve experiences that may be difficult to put into words [1,2]. In the existing literature we found consensus that arts activities offer alternative ways of engaging with end-of-life (EoL) issues, e.g., by involving all senses and including non-verbal modes of communication [1,2,3,4,5].

Arts activities have been used in healthcare settings, death education, and therapies to ease communication about EoL issues [2,3,6]. They are also used for health promotion purposes to engage communities in EoL issues, helping to encourage conversation and increase the sense of social support [2,7]. Examples of arts activities include painting, music making, dance, and pottery, with generally a focus on arts processes rather than finished products [2,5].

Such arts activities are commonly facilitated by professional artists or art therapists [1,2,8]. Along with offering alternative modes of expression, arts activities have been found to support communities, patients, families, and caregivers in coping with change, meaning-making, and personal growth [2,9,10,11,12]. Furthermore, collective arts activities have been found to offer possibilities for creating relationships and supportive communities [8,9].

It is notable that research literature in this field predominantly describes arts activities developed and facilitated by institutionalized healthcare services. There appears to be a lack of research on arts activities in community settings to support the involvement of individuals and organizations outside healthcare institutions in issues related to dying, death, and loss, here referred to as community engagement with EoL issues. Furthermore, there is little literature about the specific ways, or mechanisms through which arts activities function to support engagement with EoL issues [1]. Therefore, in this article, we aim to explore mechanisms in arts activities that support community engagement with EoL issues.

### Contextual Background: Studio DöBra

The arts activities underlying this investigation are part of Studio DöBra, a project within the DöBra research program in Sweden (“DöBra” is a pun which literally means dying well, but figuratively ‘awesome’). The DöBra research program applies a health promotion approach to engage communities in EoL issues [13]. In Studio DöBra, we partnered with community organizations to bring together children (9 y/o) and older adults (most 80+) in a series of arts workshops to engage with issues related to dying, death, and loss [4,5]. Partnerships and development of arts workshops were informed by principles of community-based participatory research [14]. However, we refer to our process as community-based participatory *action* research (CBPAR), since learning through doing, i.e., action, is central to our approach [15,16].

Two Studio DöBra iterations were developed; Studio DöBra 1 took place in 2016 in a multi-ethnic urban area outside a large city, Studio DöBra 2 in 2018 in a mid-sized town in a more rural area. These were developed by project groups consisting of first author MK, a PhD student with a design background, and the community partners shown in Table 1. Studio DöBra 1 partnerships were initiated by MK, while Studio DöBra 2 partnerships were initiated by the municipal organization for culture, based on positive word of mouth about the first iteration. As MK was part of both project groups, he contributed with lessons learned from Studio DöBra 1 in his engagement in Studio DöBra 2. Each iteration involved eight children and eight older adults in a series of five weekly, two-hour arts workshops. Table 1 summarizes characteristics of each Studio DöBra iteration as well as demographic information about those who were involved.

In line with CBPAR principles, project groups developed arts workshops through interactive cycles of action and reflection [14,15], i.e., using lessons learned to inform following workshops. Community partners with arts backgrounds took the lead in this process in each group. In Studio DöBra 1, these partners ran an artistic organization for children in the neighborhood in which they facilitated design projects, often explicitly using play as a pedagogical tool, e.g., through games and role play. In Studio DöBra 2, these partners had backgrounds in visual and performing arts, as well as primary school arts education, working together for the first time in Studio DöBra.

Partners with arts backgrounds also facilitated the workshops, with support from the other partners. During the arts workshops, participants explored topics related to dying, death, and loss through e.g., making collages in different materials, sewing, and creating sculptures. The findings provide more details about the arts activities.

## 2. Methods

### 2.1. Community-Based Data Generation

After approval by the Swedish ethical review board (2016/1517-31/5; 2018/825-32), partners, older adults, children, and their parents all signed informed consent forms. Details about the involved partners, children, and older adults, are shown in Table 1. After each workshop, partners held reflective meetings sharing their observations, reflections, and informal feedback from participants; these became the basis for developing subsequent workshops. Documentation from these reflective meetings, i.e., audio recordings and transcripts, are one form of data underlying analysis.

During the arts workshops, MK acted as participant observer, documenting observations through field notes, photographs, and audio recordings of some conversations, while also interacting with participants and partners. Within 10 weeks after each Studio DöBra iteration, follow-up interviews were conducted with partners, participating children, and older participants to inform development of future iterations as part of the CBPAR process. Interviews with the involved teachers were conducted by MK. Other partners were interviewed by researchers from the DöBra research program who were not part of the project groups. These interviews with partners (range 40–112 min, median 72 min) were held in conversational form, rather than being guided by an interview guide with predetermined questions, and focused on topics such as the experiences of developing and facilitating Studio DöBra workshops. All interviews with children and older participants were also conversation-based, focusing on their experiences of participating in Studio DöBra. In these interviews, a set of photographs from the workshops was used to support and stimulate the conversation. After Studio DöBra 1, MK interviewed children in a group (*n* = 6, 60 min) with the teacher present. After Studio DöBra 2, MK interviewed children individually (*n* = 8, range 15–37 min, median 28 min). MK interviewed older participants individually (Studio DöBra 1 *n* = 7, Studio DöBra 2 *n* = 7, range 31–118 min, median 71). All interviews were audio-recorded and transcribed.

### 2.2. Analysis

In an initial participatory analysis phase, partners from both project groups (indicated in Table 1) came together for two whole-day meetings to reflect on and document lessons learned. In doing so, a new insight was that elements of play, in a variety of forms, were found throughout all Studio DöBra arts activities, although partners from Studio DöBra 2 had not previously considered that they used elements of play. This led to curiosity as to whether play theory could assist in better understanding ways through which arts activities support engagement with EoL issues. In the analysis phase that followed, we therefore took an abductive approach inspired by Tavory and Timmermans [17]. This phase was university-based, led by MK in a process repeatedly discussed and reflected on with co-authors, a team of three professors in EoL/nursing research, sociology, and creative practices, external to the project groups. Community partners and MK stayed in contact concerning developments in the abductive analysis and in the community, which continued their process of learning together [14].

Abductive analysis aims to develop new theoretical ideas through the creative use of existing theory in relation to unexpected observations, it entails an iterative process of moving between empirical data and theoretical literature [17]. We chose this approach as it guided us in further investigating the hitherto unforeseen observation of play elements throughout Studio DöBra arts activities, by iteratively moving between Studio DöBra’s empirical data and play theory. In doing so we developed ideas about play elements as mechanisms in arts activities that supported community engagement with EoL issues.

Hamayon describes two camps in play studies [18]; one general approach to play, originating with Huizinga’s theory, first described in 1938 [19], and a second approaching play from specific contexts and disciplinary perspectives, e.g., Frissen et al. who study play in digital media [20]. Although Huizinga’s ideas about how human culture evolved from play may appear outdated and even somewhat problematic today, his definition of the characteristics of play remains classic in contemporary play studies [18,20]. In our analysis we used both Huizinga’s characteristics of play [19], and Frissen et al.’s critique and further development of this theory [20].

Our abductive analysis started with looking for “defamiliarized surprises” to use Tavory and Timmermans’ [17] term, i.e., observations in the empirical data that do not neatly fit existing theory. Thus, MK investigated if and how play elements described in the literature were expressed in the Studio DöBra arts workshops [17]. To this end, all available empirical data from partners, children, and older adults’ perspectives, were combined into one dataset for each workshop, using the qualitative analysis software NVivo. MK repeatedly read transcripts, listened to audio recordings, and examined workshop photographs. He then coded data for play elements based on the literature. Memo writing throughout this process helped to identify and reflect on surprising observations in relation to theory.

One central element of play we investigated, which then provided a basis for our continued analysis, is described by Huizinga as the “magic circle” [19]. According to Huizinga, play is distinct from “ordinary life” by taking place in temporally and spatially bounded play-spaces, e.g., playgrounds, arenas, and the boards of board games, all are “temporary worlds within the ordinary world, dedicated to the performance of an act apart” [19] (pp. 9–10). A magic circle is the temporal and spatial boundary that separates play from ordinary life [19]. Inside the magic circle, “the laws and customs of ordinary life no longer count” [19] p. 12. The term is widely adopted in play studies [21]. However, Frissen et al. argue that Huizinga’s theory is contradictory as it claims that play only happens outside ordinary life while it also states that play is interwoven into many parts of our daily lives [20]. Frissen et al. therefore assert that players can be in and outside playful modes simultaneously [20].

In our data we could identify a Studio DöBra magic circle, i.e., the spatial and temporal boundaries of the arts workshops. Additionally, in line with Huizinga, the norms and customs inside the Studio DöBra magic circle deviated from ordinary life in that participants and partners interacted across generations beyond their families and engaged with EoL issues, whereas this would not occur in ordinary life in the same way, as indicated by our previous research [4,5]. However, participants and partners appeared to move in and out of playful modes within the Studio DöBra magic circle, rather than Huizinga’s idea of a magic circle as a separation between play and ordinary life, or Frissen et al.’s ideas about play and ordinary life occurring simultaneously. Through iterative analysis, moving between data and theory, we can describe the functionality of the Studio DöBra magic circle in supporting engagement with EoL issues.

Other play elements we investigated are characterized by ambiguity, as pointed out by Frissen et al. [20]. They state for example that play includes both “freedom and force”, because it requires voluntary participation, but also adherence to rules [19,20]. Play also relates to both “reality and appearance”, as it is part of ordinary life, but also characterized by players’, often unspoken, consciousness of their play as different from ordinary life, e.g., just pretending [18,20]. Furthermore, play is both an “individual and collective” activity, requiring players’ individual attention [20], but often leading to “the formation of social groupings” [19].

However, during analysis we noted a friction between our empirical data and the theoretical play elements. Rather than using force, we found that partners were balancing freedoms with restrictions in the ways they facilitated arts activities. We found that participants and partners dealt with imagination and real-life experiences, rather than appearance and reality. Additionally, we found that when partners and participants moved in and out of playful modes, they did so individually, in sub-groups, or collectively. As we went back and forth between theory and data, we further developed play elements and identified ways in which they supported engagement with EoL issues in Studio DöBra arts activities.

We continued analysis by searching for variation in our modified play elements throughout the data [17]. As the use and functionality of play elements changed across and within workshops, further analysis focused on what led to these changes. Specific events within the arts activities were identified as particularly illustrative of these changes. To investigate these in detail, MK transcribed relevant parts of workshop audio recordings and drew from play theory to interpret them. He also generated illustrations from photo-documentation, which he combined with interview/workshop transcripts, to visualize different perspectives on events, as shown in the findings. Continued memo writing helped in formulating findings.

Lastly, MK met virtually (due to COVID-19-related restrictions) with the partners who participated in the initial participatory analysis to discuss tentatively formulated findings. Partners confirmed the findings from their perspectives, but also provided feedback which added nuance to the final formulation.

## 3. Findings

We use our modification of play elements from theory, to explain mechanisms supporting community engagement with EoL issues in arts activities. We identified four mechanisms: Creating permeable magic circles; Balancing restrictions and freedoms; Approaching dying, death, and loss through imagination and real-life experiences; and Continuing a sense of community after ending the arts workshops. In presenting our findings, we refer to examples from Studio DöBra arts activities. We chose to discuss one exemplar from each Studio DöBra iteration more in depth throughout the findings to demonstrate the diverse ways in which mechanisms acted in each iteration, and to illustrate ways in which various mechanisms acted in concert.

The Studio DöBra 1 exemplar comprises events from an arts workshop dealing with the question “Where do we end up after we die?” Participants explored this using travel as a metaphor for dying and death, creating descriptions of the afterlife as if it were a travel destination, and then building a vehicle to play-travel there. The Studio DöBra 2 exemplar consists of related events from the last three workshops of this iteration, revolving around a dead bird, brought to the workshops by a partner. Using sewing tools and fabrics of various colors and patterns, participants created images symbolizing the bird’s life, death, and afterlife to decorate a large fabric sheet. During the final workshop, partners and participants held a funeral ceremony for the bird.

We use empirical data to illustrate findings. For confidentiality, partners and participants are given pseudonyms and personal information is omitted.

### 3.1. Creating Permeable Magic Circles

We defined the Studio DöBra magic circle as the spatial and temporal boundaries of the arts workshops. Studio DöBra 1 workshops were held in different places—a children’s library, an activity center for older adults, and at the artistic organization for children. The Studio DöBra 2 workshops all took place in a project room already used for weekly creative sessions by the participating older adults. All locations were closed to the public during workshops, creating spatial and temporal boundaries from ordinary life.

**Figure 1:**
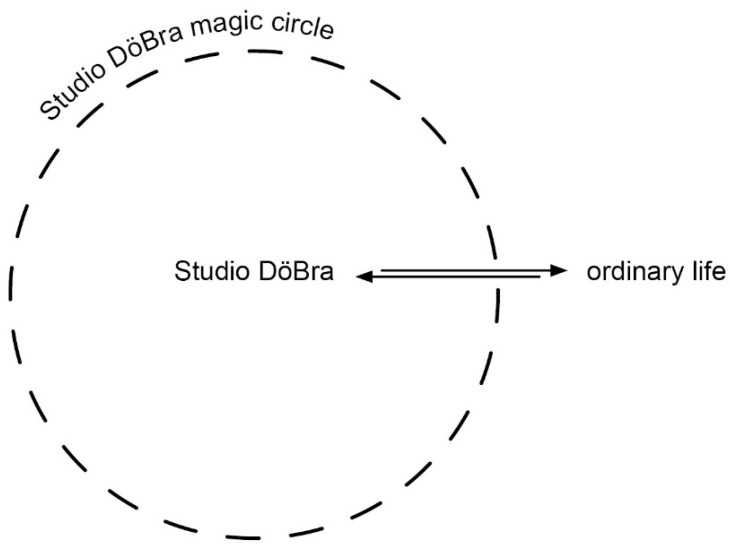
The Studio DöBra magic circle as a permeable boundary.

The Studio DöBra magic circle provided the context for intergenerational interaction and engagement with EoL issues. Within this, partners and participants sometimes used their imagination to engage with EoL issues, and sometimes introduced elements from ordinary life by sharing EoL-related experiences, such as the death of a family member or a pet. Additionally, our earlier analyses indicated that participants created spaces in their social networks for engaging with EoL issues stimulated through Studio DöBra participation [4]. We therefore conceptualize the Studio DöBra magic circle as a permeable boundary through which participants could introduce experiences from ordinary life into Studio DöBra and introduce elements from Studio DöBra into their ordinary lives, as illustrated in Figure 1.

In some events, smaller groups of participants seemed to gather into their own magic sub-circles. In the Studio DöBra 2 exemplar, the whole group discussed how birds are born from eggs and how the bird at the workshop might have died, e.g., by falling as it was trying to fly. A smaller group of children began fantasizing among themselves, wondering if the bird was born from an Easter egg, killed by a meteor, and then transformed into a giant kiwi. During the reflective meeting which followed, partners discussed this:

Sandra: *I thought it was interesting how* [I] *reacted when it got silly, because I noticed how I reacted myself* […] *In my role, I thought, “oh, now maybe the older people take offense that it’s not a respectful way to talk about death, but that it becomes…”*

Anna: *I think that I took a little offense, or I thought that they weren’t… weren’t really present.*

Sandra: *Yes, that they were somewhere else.*

Anna: *They were in their play…*

Older adults reflecting on this in follow-up interviews did not appear to have taken offense, although some questioned children’s understanding of the seriousness of death as illustrated by Berit (older participant):

*They don’t have the experience* […] *they’re still direct. You could hear it* [when they talked about] *the kiwi and everything else they came up with, which is so delightful with children.*

Thus, events in which a group of participants segregated themselves into a magic sub-circle could create tensions when outsiders perceived the participants to be “not present” or if the play was interpreted as being not serious enough in relation to EoL issues. However, segregating into magic sub-circles may be a way for participants to engage with EoL issues in their own way. An unresolved challenge for partners was therefore the degree to which they should steer participants’ engagement with EoL issues in response to these tensions. They tried different approaches as described below.

### 3.2. Balancing Restrictions and Freedoms

Studio DöBra arts activities were guided by partners trying to balance restrictions and freedoms. This occurred in part through the use of questions and topics. In the Studio DöBra 1 exemplar, descriptions of the afterlife destinations were based on predetermined questions, e.g., What language is spoken? and what sights are worth seeing? Restricting the questions seemed to give partners a sense of control over the EoL issues raised by participants. However, partners sometimes wondered if these restrictions inhibited participants from exploring their own questions. In a follow-up interview, Charlie (partner) proposed “You might ask questions that are a little more open… but then maybe it becomes less playful,” thus suggesting that increasing freedoms might decrease playfulness. In another Studio DöBra 1 workshop, participants were asked to create alternative ways to measure time. Partners had assumed that participants would relate the passing of time to the EoL, but later reflected that this activity did not trigger the intended discussions. Thus, narrowly defined questions could restrict participants from freely exploring, whereas more open formats risked failing to initiate engagement with EoL issues.

Based on lessons learned from Studio DöBra 1, Studio DöBra 2 partners tried to achieve balance using the first workshop for participants to freely determine the EoL issues they wanted to address. Partners met with the children and older adults in separate groups in advance. Each group made a mind-map of their thoughts about dying, death, and loss. In smaller intergenerational groups in the first joint workshop, participants compared these mind-maps and together chose words or phrases as topics for collages. By relinquishing control over the topic selection, partners also shifted the responsibility for choosing topics to participants.

Another issue concerned the artistic processes and products, which were envisioned and preplanned by partners. Some partners and participants reflected on some of the arts activities’ processes as not creating enough space for self-expression. Siv (older participant) explained:

*I wish we’d had more time to talk* [about EoL topics] *and less time to do arts and crafts. Because that took some of the focus.*

In the Studio DöBra 2 exemplar, partners envisioned that the whole fabric sheet would be filled with participants’ images symbolizing the bird’s life, death, and afterlife. However, they noticed that participants were creating small images that would not fill the fabric in the available time. Partners therefore told participants to create larger images, to which Alva (child) responded:

*“But we need to have respect for the bird, it cannot become ugly.”*

Alva’s remark pointed out that partners’ expectations did not reflect her efforts to show respect in her own way. Partners thus controlled processes and products by imposing restrictions, which in this case were not aligned with participants’ processes and expectations.

In reflective meetings, partners identified such tensions, and in response adjusted planning of following arts activities to try to better balance restrictions and freedoms. Partners also adjusted approaches during arts activities, as the following excerpt from the Studio DöBra 1 exemplar illustrates. Here, Sasha (partner) adjusted freedoms and restrictions to stimulate participants to play-travel to the afterlife destinations they had described, with the vehicle they had built.

Sasha (addressing participants): *It could be that I would decide, but* […] *now you have this vehicle* […] *and three* [afterlife destinations] *to travel to* […] *so how are you going to travel there? Do you have any idea? This is a little bit like playing.*

[Participants have a lively discussion but seem uncertain about what to do.]

Sasha: *Everyone who built this, now you are going to test traveling to these three different destinations in your imagination* […] *We’re in a kind of airport now. How are you going to do this?*

[Participants discuss but still cannot agree.]

Sasha: *Shall we let each group take the others to their destination? You understand? Yes or no?*

[No clear response.]

Sasha: *OK, I’ll make it simple. There are three groups, and each group decides how you travel to your destination. I decide that this group (points to one group) starts. You’re the pilots, the others are the passengers.*

[Participants get ready.]

Sasha: *Everyone! Let’s pretend this is a theatre or a film set, and we say ‘ACTION!’ so the film starts, the event of traveling to this destination.* […]

[Participants get ready.]

Sasha: *OK!* […] *ACTION!*

**Figure 2:**
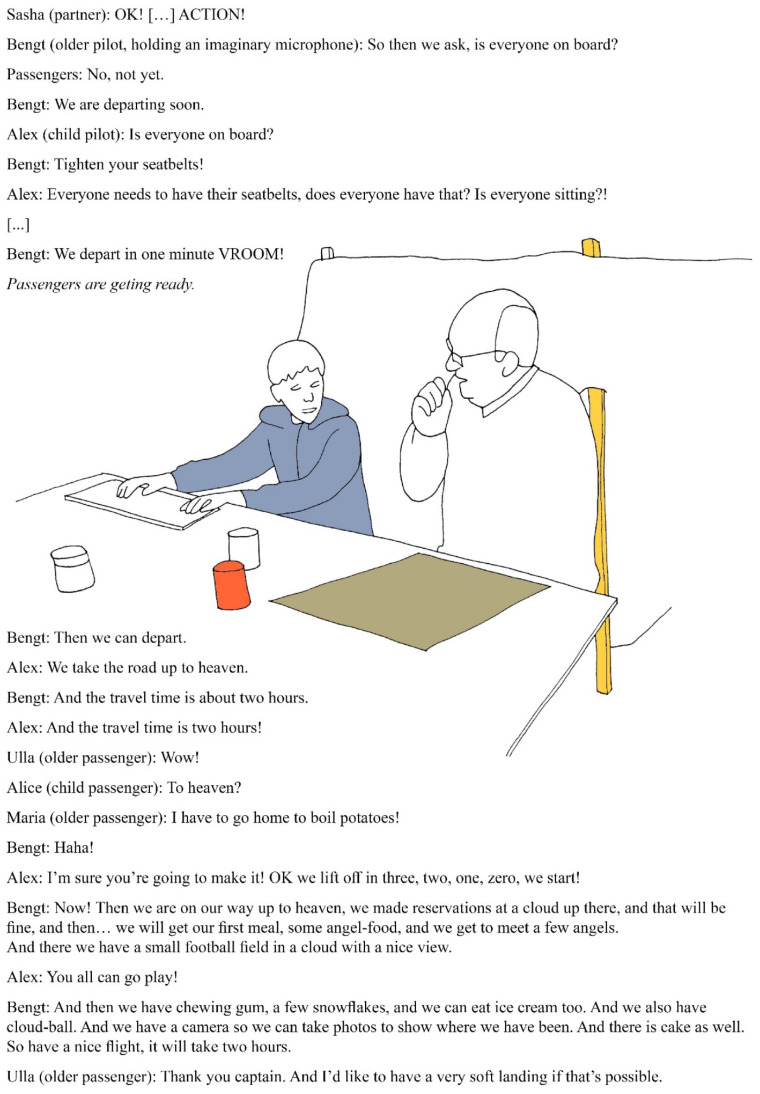
Traveling to after life (illustration by author MK, based on a photograph of the workshop).

As participants seemed uncertain about what to do and could not agree, Sasha gradually decreased free choice by increasing restrictions. Sasha thus created a magic sub-circle by transforming the space into an imaginary airport and film set and marking temporal boundaries by saying “ACTION!” This led to children and older adults exploring different afterlife-scenarios together, as illustrated in Figure 2.

Although partners interpreted the event illustrated in Figure 2 as play, participants reflecting on this arts activity in follow-up interviews did not use this term, but rather described what they did, as illustrated by the following two excerpts:

Nils (child): *We got to build a vehicle, and then we got to sit in it and travel* […] *to different* […] *after-death-places.*

Bengt (older participant): *We talked about… up in heaven* […] *we traveled up there with the spaceship… I said* […] *it will take two hours maybe to travel there, and that’s when* […] *we came to the conclusion that there would be food* […] *and then the little girl said that chewing gum was important.*

When restrictions and freedoms were well-balanced, partners reflected on participants being independent in their execution of the arts activity and engagement with EoL issues:

Anna (partner): *We (referring to partners) all felt as if we weren’t really needed, because they were so independent, both in… that they understood, that the assignments were clear, I think, and that they worked well together, the older adults and the children, and that the material was there so they helped each other technically and theoretically.* […] *and that was a nice feeling. Then it felt like… we had prepared well, when we weren’t needed.*

In conclusion, restrictions in aspects of arts activities enabled participants to explore EoL issues, but also risked diminishing both their agency and artistic freedom. Too much freedom could leave participants uncertain about both their roles and what was expected of them. Partners tried to find balance by creating a magic circle with a minimum of restrictions within which they felt in control and at the same time provided participants with enough freedoms and agency to engage with EoL issues on their own terms.

### 3.3. Approaching Dying, Death, and Loss Through Imagination and Real-Life Experiences

Some partners reflected on finding it challenging to approach EoL issues, as death is part of our reality but at the same time abstract and difficult to grasp. Some partners described feeling insecure about facilitating arts activities addressing questions to which they do not have answers themselves. Additionally, as EoL-related experiences and beliefs are personal, project groups aimed to create a space which could contain and foster diverse perspectives. Partners therefore approached EoL issues in arts activities through a combination of imagination and real-life experiences.

The first project group often used metaphors as a means to call on participants’ imaginations to explore EoL issues, e.g., travel as a metaphor for dying and death in the Studio DöBra 1 exemplar illustrated in Figure 2. The use of metaphors could blur the line between imagination and real-life in arts activities. Participants could, for example, use metaphors to talk about personal EoL experiences, questions, and beliefs, as in the following conversation from the Studio DöBra 1 exemplar, involving a small group of participants and partners:

Alex (child): *My mother says that my grandmother has gone on a trip, but she’s dead.* […] *She still says that she’s gone on a trip* […] *and I don’t believe her.*

Sasha (partner): *How long has she been gone?*

Alex: […] *since I was three.*

[…]

Bengt (older adult): *It’s hard on kids, they ask, where did they go* […] *it can be sensitive too… She’s not around anymore.*

MK: *It’s strange.*

Bengt (older adult): *It’s strange. You grow up with your family, you can think that they’ll be there forever and then they’re gone, that’s the way of life, the same with flowers in the field, during the summer there are beautiful flowers and then they die, after that there’ll be new ones, those can be their children and grandchildren, the same with trees, everything. (Addressing Alex) Your mother gave birth to you, when you grow up you’ll have children, that’s how we are.*

Later, a partner overheard Ulla (older participant) say to the children that she would, perhaps soon, send them a postcard from the afterlife destination they had just described together. Thus, it seemed that metaphors were used to talk about personal EoL experiences, questions, and beliefs explicitly, as in the excerpt above, or implicitly, as with Ulla who seemed to indirectly convey her awareness of nearing the end-of-her-life.

In contrast, in the Studio DöBra 2 project group chose to approach the topic head-on by introducing the dead bird. Partners recognized that as adults they had expected death to be discussed seriously and wondered how they could create space in which everyone has respect for one another’s perspectives. The final Studio DöBra 2 workshop revolved around a burial ceremony for the dead bird. To create a serious and respectful atmosphere, partners changed some elements of the magic circle. A stronger sense of coming into the magic circle was created by keeping participants from entering the project room as they arrived, entering together instead. The table which usually displayed arts materials, now showcased red roses, a candle and a little box as a coffin for the dead bird. The other tables were covered with the fabric decorated with the images of the bird’s life, death, and afterlife, sewn by participants. As participants came into this space, they were quiet and seemed moved, as exemplified by Siv (older participant):

*That was very beautiful, very beautiful.* […] *The pretty fabric there and everything was so pretty.*

Jenny (partner) said:

*It became… very respectful,* […] *calm, and harmonious when everyone went in together,* […] *and the project starts, and then we leave other things outside.*

**Figure 3:**
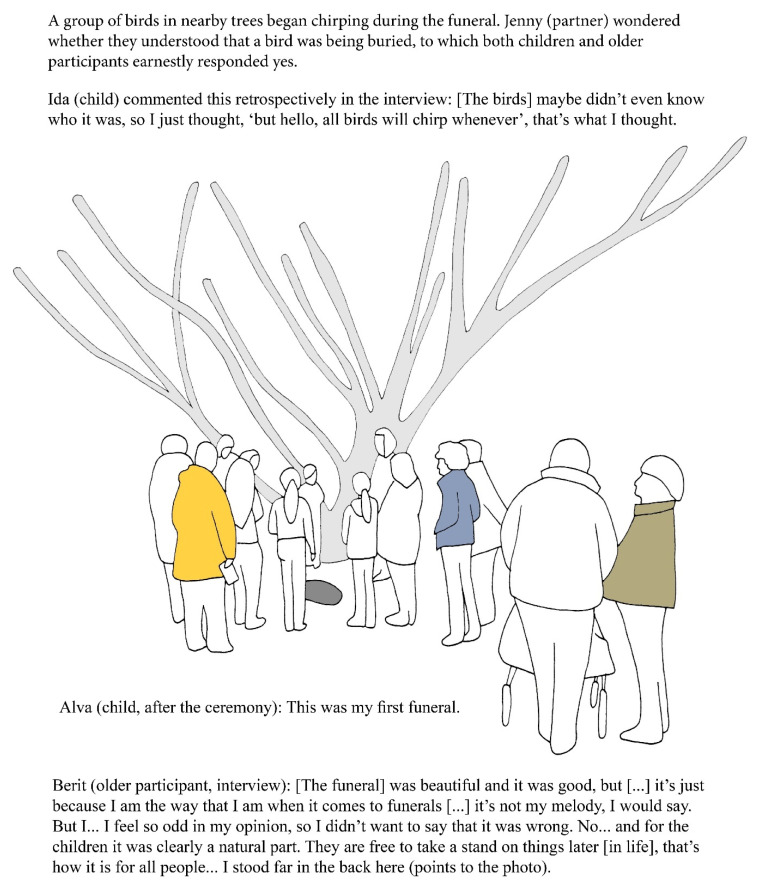
Funeral for a bird (illustration by author MK, based on a photograph of the workshop).

Partners planned the ceremony to include elements commonly found in Swedish funerals. Participants wrote farewell notes to the bird, after which they went outside to bury the bird under a tree and left flowers, as illustrated in Figure 3.

Quotes from participants in Figure 3 show that different perspectives about the funeral for the bird co-existed, but that not everyone shared their perspective openly. It seemed that some participants self-censored as they anticipated that their perspective might be deviant, as exemplified by Ida (child) and Berit (older participant). Furthermore, the line between imagination and real-life experiences became blurred, as shown by Ida (child) who seemed to perceive the group as using imagination, and Alva (child) and Berit (older participant) who described the ceremony as a real funeral. There appears to be a difference between the way imagination is used in this exemplar compared to the Studio DöBra 1 exemplar. Perhaps this is due to differences in the magic circle which in the Studio DöBra 1 exemplar was designed by partners to initiate a playful engagement with EoL issues using metaphors, whereas in the Studio DöBra 2 exemplar, partners designed the magic circle to create a serious and respectful atmosphere using elements of real-life funerals.

### 3.4. Continuing a Sense of Community after Ending the Arts Workshops

As noted above, according to Huizinga play is characterized by the formation of a “play-community”. This community tends to “stress their difference from the common world by disguise or other means” [19] p. 13. We found examples of this in both Studio DöBra iterations as certain events became symbols only understood by participants and partners. During the first Studio DöBra 1 workshop, participants and partners created and played a new form of Bingo. ‘Bingo-death’ became a defining symbol for Studio DöBra 1 as it was referred to throughout the workshops and interviews, with some saying they found it hard to express its’ meaning to outsiders. During the first Studio DöBra 2 workshop, Emil (child) created an image of a flying pig for a collage about dying, and explained that he believed that when he dies, a flying pig will bring him to heaven. The image of the flying pig became symbolic for Studio DöBra 2, as participants and partners referred to it throughout workshops and interviews.

The tangible products of the arts activities were another factor bonding participants. Participants brought up the question of ownership by asking whether they could take the objects home. In Studio DöBra 1, partners explained that participants were collective owners, and that products would be saved in an archive to which participants would have access. In Studio DöBra 2, products were shown at the children’s school, which then discarded them without further consultation.

During the last Studio DöBra 1 workshop, participants created an exhibition at the children’s library, displaying their products accompanied by their texts and collages of workshop photographs, after which they role-played its’ opening event. The day after, outsiders were invited to the formal opening of the exhibition. The funeral for the bird was also conceptualized as a goodbye to Studio DöBra 2. Alva (child) not only wrote a farewell note to the dead bird, but also to Studio DöBra, which she left in the project room:

*“It was a lot of fun to participate* [in Studio DöBra]*! It was a pity that the bird died because it could have lived longer and done more in life!”*

After the ceremony, a group of guests were invited to an event during which participants showed what they had created. While older adults said they had no-one to invite, children invited family and friends, and partners invited colleagues and journalists. Thus, the conclusions of both Studio DöBra iterations were marked by a final collective activity, after which the magic circle was opened to outsiders.

Although the temporal and spatial structures of the Studio DöBra magic circle ended at the conclusion of each iteration, reflections in follow-up interviews suggest that a sense of community continued to exist in various forms. This is in line with Huizinga’s ideas about the continuation of play-communities: “The feeling of being ‘apart together’ in an exceptional situation, […] and rejecting the usual norms, retains its magic beyond the duration of the individual game” [19]. Partners and participants from both Studio DöBra iterations talked about continuing to meet in passing in the neighborhood [4]; Studio DöBra 2 participants also talked about visiting the bird’s grave. The Studio DöBra 1 exhibition was later shown at the activity center for older adults, and partners organized a reunion two months later.

## 4. Discussion

We aimed to explore mechanisms in arts activities that support community engagement with EoL issues, through investigating Studio DöBra arts activities. Based on the partners’ unexpected observation of play elements throughout the arts activities, we continued analysis with an abductive process using play theory. In doing so we modified theoretical play elements to explain mechanisms supporting engagement with EoL issues in arts activities. We found four mechanisms: (1) Creating permeable magic circles. The Studio DöBra magic circle provided the context for engagement with EoL issues, with participants sometimes segregating into magic sub-circles within which they engaged with EoL issues in their own ways. Partners struggled with the degree to which they should steer participant’s engagement with EoL issues. (2) Partners aimed to balance restrictions and freedoms in arts activities to support participants’ independent engagement with EoL issues and execution of arts activities, while also creating a sense of control for themselves. (3) Partners and participants approached EoL issues through both imagination and real-life experiences to deal with the potential sensitivity of the topic, but also to foster different perspectives of EoL-related experiences and beliefs. The line between imagination and real-life was not always clear in arts activities. (4) Continuing a sense of community after ending the arts workshops. We found indications of a sense of community developing through arts activities, and remaining after Studio DöBra ended, which supported continued engagement with EoL issues.

Although most literature about supporting EoL conversations through arts activities derives from healthcare, therapy, or community settings with ill or bereaved individuals [2,3,10], Studio DöBra was community-based and deliberately involved participants who were not imminently dying, in an effort to support early engagement with EoL issues. Our findings thus complement this literature with insights into mechanisms supporting engagement with EoL issues in community-based intergenerational arts activities. Our study has potential to contribute to theory-building that can inform arts activities supporting community engagement with EoL issues. One limitation of this abductive qualitative study is that it is based on play theory only. We suggest therefore that further research may not only build on our findings by critically applying mechanisms in the development and facilitation of arts activities to support community engagement in EoL issues, but might also draw on additional theories for further understanding.

In line with findings from Studio DöBra, literature points to the potential of arts initiatives and intergenerational programs to promote a sense of community [4,22,23,24]. It is noteworthy that the Studio DöBra magic circle shares features with McMillan’s description of elements which promote a sense of community [25]. The Studio DöBra magic circle defines spatial and temporal boundaries, which McMillan points out as being essential for establishing a space in which individuals can identify as a group. McMillan contends that the community should provide a space in which members feel safe to share personal experiences [25]. This appeared to be occasionally challenging in Studio DöBra, as some participants seemed to self-censor when they felt their perspective was outside the norm. Self-censoring on the other hand, might also point to participants’ agency in modulating their engagement with EoL issues [4].

EoL experiences may be considered ‘unspeakable’ as they are often sensitive and difficult to put into words [1]. The Studio DöBra magic circle provided time, space, and permission for engaging with EoL issues, thereby being responsive to participants’ desire for spaces in which to talk about these issues [4,26]. While talking was part of engaging with EoL issues, partners and participants also engaged through acting, doing, and making, consistently using both imagination and real-life experiences.

During the initial participatory analysis phase, partners expressed surprise at the older participants’ child-like playfulness in some arts activities, as in the Studio DöBra 1 exemplar. Partners wondered whether interaction with children may have helped catalyze this; however, our data does not allow for definitive conclusions. Hamayon argues that play in Western cultures is commonly seen as restricted to childhood and adult leisure [18]. Thus, partners’ interpretation of older participants’ play is likely to be influenced by these cultural norms. This implies that cultural norms may constrain the kinds of play adults engage in. It may be that intergenerational interaction lowered the threshold for child-like play. This is in line with the idea of a magic circle within which norms differ from ordinary life [19]. However, it remains unclear as to whether older adults would adopt similar playful engagement with EoL issues in the absence of children. Furthermore, gender roles may also affect older adults’ engagement in arts activities with play elements; however, systematic investigation of this is beyond the scope of this study. These notions also point to a need to further consider cultural and gender norms regarding play and talking about EoL issues, when considering transferability of our findings to other settings [27].

One strength of this study is that it is based on data triangulating perspectives from partners, children, and older adults [28]. It is important to reiterate that the observations regarding play elements in the arts activities were made by partners. Participants did not use the term play as they reflected on their Studio DöBra participation in follow-up interviews. Thus, rather than conceptualizing all arts activities as play, we used play theory to investigate mechanisms in arts activities that support engagement with EoL issues. As the abductive analysis phase was led by researcher/partner MK, one role of co-authors as researchers external to Studio DöBra, was to support MK in critically reflecting on his own roles as partner and researcher.

Another strength of this study is the triangulation of different types of qualitative data [28], i.e., follow-up interviews, reflective meetings, and participant observations including photographs, audio recordings of conversations, and MK’s field notes. As it was not always possible to produce high-quality audio recordings during arts workshops, other types of data were also used to analyze some of the verbal interaction, e.g., MK’s fieldnotes and partners’ reflective meetings. However, this may risk skewing the interpretations towards partner perspectives. Prior to the start of Studio DöBra, one partner expressed concerns that MK’s participant observation methods, e.g., audio-recording conversations and photographing, might disturb participants’ interaction and engagement, thereby risking compromising the magic circle. MK considered himself an insider in the magic circle through his role as partner, but others may have considered him an outsider due to his research activities. On the other hand, this method itself is in line with our conceptualization of the magic circle as a permeable boundary, allowing MK to be both participant and observer simultaneously.

Our findings contribute to understanding mechanisms in arts activities supporting community engagement with EoL issues. The notion of the Studio DöBra magic circle can help to understand ways in which other health promoting initiatives can provide time, space, and permission to engage with EoL issues in community contexts. By applying our modified play elements to arts activities aiming to support community engagement with EoL issues in other contexts and with other groups, we hope that researchers and practitioners can further explore these mechanisms and build on our findings. In Studio DöBra, partners adapted their approaches in response to tensions they observed and reflections regarding their own roles. Reflective practice [14], e.g., gathering and reflecting on informal feedback from participants during arts activities, and reflective meetings in conjunction with workshops, supported partners in this process. We therefore suggest that transferring our findings to different contexts should be coupled with continuous reflective practice to adapt approaches sensitive to the local context and relevant for participants.

## Figures and Tables

**Table 1:** Characteristics of the two Studio DöBra iterations and demographic information.

**Studio DöBra 1, 2016**			
	**Project Group Partners**	**Children**	**Older Adults**
	*n* = 7 DöBra research program: MK ^a,b^ Artistic organization for children: Artist manager ^a,b^ Artist artistic director ^a,b^ Freelance artist ^a^ Activity center for older adults: Activity manager ^a,b^ Children’s library: Librarian After-school center: Teacher	*n* = 8	*n* = 8
Invitation to participate	MK approached community organizations	Through after-school center and parents	Individually, through involved community organizations
Gender	5 women, 2 men	4 girls, 4 boys	5 women, 3 men
Age	Median 37, ages 28–65	9	Median 82 ages 65–85
**Studio DöBra 2, 2018**			
	**Project Group Partners**	**Children**	**Older Adults**
	*n* = 7 DöBra research program: MK ^a,b^ Municipal organizations for culture: Producer of cultural activities ^b^ Producer of cultural activities for older adults ^a,b^ Freelance artist ^a,b^ Municipal organization for elder care: Activity manager ^a,b^ After-school center: Teacher	*n* = 8	*n* = 8
Invitation to participate	Municipal organization for culture approached MK and the other community organizations	Through after-school center and parents	As a group (independently hosting weekly creative sessions), through the municipal organization for elder care
Gender	6 women, 1 man	4 girls, 4 boys	8 women
Age	Median 33, ages 29–64	9	Median 83.5 ages 66–93

^a^ Partners with an arts background. ^b^ Partners who participated in the participatory analysis phase. The other partners did not participate due to personal circumstances or lack of professional mandate.

## Data Availability

For original data, please contact the corresponding author; ethical approval does not cover making data openly accessible.
